# Conspecific Sperm Precedence Is a Reproductive Barrier between Free-Spawning Marine Mussels in the Northwest Atlantic *Mytilus* Hybrid Zone

**DOI:** 10.1371/journal.pone.0108433

**Published:** 2014-09-30

**Authors:** Lara K. J. Klibansky, Michael A. McCartney

**Affiliations:** Department of Biology and Marine Biology, University of North Carolina Wilmington, Wilmington, North Carolina, United States of America; Simon Fraser University, Canada

## Abstract

Reproductive isolation at the gamete stage has become a focus of speciation research because of its potential to evolve rapidly between closely related species. Conspecific sperm precedence (CSP), a type of gametic isolation, has been demonstrated in a number of taxa, both marine and terrestrial, with the potential to play an important role in speciation. Free-spawning marine invertebrates are ideal subjects for the study of CSP because of a likely central role for gametic barriers in reproductive isolation. The western Atlantic *Mytilus* blue mussel hybrid zone, ranging from the Atlantic Canada to eastern Maine, exhibits characteristics conducive to the study of CSP. Previous studies have shown that gametic incompatibility is incomplete, variable in strength and the genotype distribution is bimodal—dominated by the parental species, with a low frequency of hybrids. We conducted gamete crossing experiments using *M. trossulus* and *M. edulis* individuals collected from natural populations during the spring spawning season in order to detect the presence or absence of CSP within this hybrid zone. We detected CSP, defined here as a reduction in heterospecific offspring from competitive fertilizations *in vitro* compared to that seen in non-competitive fertilizations, in five of the twelve crosses in which conspecific crosses were detectable. This is the first finding of CSP in a naturally hybridizing population of a free-spawning marine invertebrate. Our findings support earlier predictions that CSP can promote assortative fertilization in bimodal hybrid zones, further advancing their hypothesized progression towards full speciation. Despite strong CSP numerous heterospecific fertilizations remain, reinforcing the hypothesis that compatible females are a source of hybrid offspring in mixed natural spawns.

## Introduction

Conspecific sperm precedence (CSP) is a post-mating, pre-zygotic reproductive barrier and is defined as a non-random increase in fertilization success of conspecific sperm when an egg is challenged with both conspecific and heterospecific sperm simultaneously [1]. The interaction of gametes was initially ignored in the study of reproductive barriers, however, recent studies in animal and plant systems have revealed great potential for a sizable role in the speciation process [2], [3]. With this new appreciation of the relevance of CSP, research has begun to focus on its operation within hybrid zones. Hybrid zones occur where genetically distinct populations meet, mate and produce mixed-ancestry offspring [4], and offer an opportunity to explore how reproductive barriers, such as CSP, may operate in a population in which divergent lineages have made secondary contact and some argue [5], [6] are in a transitional phase of progress towards speciation.

CSP has been demonstrated to be an effective barrier in a number of terrestrial hybrid zones, and more recently, in free-spawning marine invertebrates [1], [7], [8]. Terrestrial organisms often have complex courtship and mating behaviors that can complicate assessing the role of gamete interactions in prezygotic isolation. In contrast, free-spawning marine invertebrates have few, if any, mating-associated behaviors, with the exception of possible prespawning chemical cues and aggregative behaviors at spawning [9]. Given this short list of candidate prespawning isolating barriers, interaction and competition between conspecific and heterospecific gametes may be crucial to reproductive isolation in mixed-species populations.

Two studies have documented CSP in free-spawning marine invertebrates, although in neither case are hybrids a feature of natural populations. Geyer and Palumbi [8] documented reciprocal CSP in two species of sea urchin, *Echinometra oblonga* and *E*. sp C. A second study by Harper and Hart [7] documented CSP in *Asterias* sea stars. Both studies employed laboratory crossing experiments, where sperm from two males were combined and mixed with eggs from one female. Though these species are found to produce viable hybrids in a laboratory setting, there is no reliable evidence of hybrids in natural populations, in either case. In both studies the results indicate that though CSP is present between the two species, it is incomplete. Since reproductive isolation in natural populations in these cases is effectively complete, the importance of partial CSP in the speciation process is unclear. In an effort to more directly evaluate the role of CSP as an isolating mechanism, we have chosen to examine it in a natural hybrid zone between two populations where reproductive isolation is present, but incomplete.

*Mytilus* blue mussels are marine broadcast spawners with a worldwide distribution, forming hybrid zones wherever the species overlap geographically [10]. There are two different *M. trossulus/M. edulis* hybrid zones located on opposite coasts of the Atlantic Ocean. In addition to the northwestern Atlantic hybrid zone, the focus of this paper, which ranges from northern Newfoundland to the eastern-most coast of Maine, there is the Baltic *Mytilus* hybrid zone. Both of these zones are the products of recent, secondary contact between species, however, they exhibit very different frequency distributions of hybrid genotypes. The Baltic hybrid zone is unimodal, being composed mainly of hybrids with each intermediate hybrid class being about equal in frequency, including F_1_, F_2_ and a range of backcrosses, along with some pure *M. edulis*. There are, however, no pure *M. trossulus*. In contrast, the northwestern Atlantic hybrid zone exhibits a bimodal distribution of hybrid genotypes. F_1_ and other intermediate hybrids are less frequent, but still represent approximately 10–26% of the population, and the pure parental individuals are most common [11]. This bimodal distribution suggests that the two *Mytilus* populations composing the northwestern Atlantic Mytilus hybrid zone are progressing toward complete reproductive isolation [6]. The partially-formed-but-incomplete reproductive isolation of these two *Mytilus* species is noteworthy because, as mentioned above, unlike most other free-spawning marine species used in studies of CSP, *M. trossulus* and *M. edulis* hybridize in nature.

Two studies, Rawson et al. [11] and Slaughter et al. [12], studied gamete incompatibility *in vitro* between *M. edulis* and *M. trossulus*. These studies were conducted in a non-competitive format by mixing the eggs of a single female with varying dilutions of sperm from a single male, carefully quantifying compatibility of all possible male/female combinations, and comparing the results for both intraspecific combinations with those from the two reciprocal interspecific crosses. Both studies clearly demonstrated stronger gamete incompatibility of interspecific crosses. What is not clear is the extent to which this gametic incompatibility blocks gene flow in natural spawning events. Spawning seasons of the two syntopic species coincide, as indicated by overlapping gametogenic cycles [13] and seasons of larval release [McCartney and Yund, unpublished]in eastern Maine. Rawson et al. [11] and Slaughter et al. [12] each showed high interspecific fertilization in some crosses involving *M. edulis* females, suggesting these to be the route through which introgression occurs in the hybrid zone. However, missing are laboratory experiments that more closely model natural spawns, in which eggs are exposed to sperm mixtures where CSP may operate. These experiments are the focus of the present study.

## Material and Methods

### (a) Specimen Collection

Adult mussels were collected from three locations in Cobscook Bay, ME: Pirate's Cove (44°50′49.70N 67 ° 01′00.71W), Leighton Point (44 ° 53′55.15N 67 ° 06′41.74W), and South Lubec (44 ° 48′56.90N 66 ° 59′10.81W). All mussels were collected under Special License Number ME 2011-41-01 issued by the State of Maine. No other specific permissions were required for any of the collection sites and neither species collected are endangered or protected. These sites were chosen based on past observations that they reliably produce animals that respond well to spawning induction, and based on the relative frequency of parental species and hybrids at these sites in prior years. Pirate's Cove is a population with a greater frequency of *Mytilus trossulus* than other Cobscook Bay sites, while at South Lubec there is a bias towards *M. edulis* [McCartney unpublished]. Mussels were removed without regard to their location in mussel beds and transported back to the Freidman Field Station (Suffolk University) where they were placed into flowing seawater tanks at ambient temperature (approximately 9 to 11°C over the time span of these experiments).

### (b) Spawning

Spawning was induced using a modified hydrogen peroxide treatment [14], [15] and gametes were processed by methods similar to those described in Slaughter et al. [12]. Individual mussels were first placed into cups containing approximately 200 ml of aged seawater (ASW) at ambient seawater temperature. Then, 1 ml of 1 M Tris HCl buffer and 120 µl of 30% hydrogen peroxide were added to the cups. Mussels were soaked for approximately 1 hour before the induction solution was poured off and 200 ml of fresh ASW was added to the cups. Mussels were observed throughout the entire process for spawning. A second induction treatment was administered if spawning was not observed approximately one hour after the first treatment. Any unspawned mussels were discarded.

When sperm release was observed, the male was quickly removed from the ASW, wrapped in a damp paper towel and stored on ice until they were strip-spawned (later in the day, when eggs were released) by making an incision in the mantle and pressing to start the flow of sperm. “Dry sperm” (concentrated sperm obtained in this manner) were collected in 1.5 ml microcentrifuge tubes and stored over ice. For later genetic identification of crossed individuals, mantle tissue was removed from the male and placed into salt saturated DMSO preservative [16] and stored at 5–9°C. Sperm counts were made using a 10-3 dilution of sperm from each male and a Neubauer hemocytometer. Dry sperm concentrations were determined and used to calculate the sperm concentrations used in both the competitive and non-competitive crosses (described below).

Once females were observed spawning, they were moved to clean ASW and allowed to continue releasing eggs until spawning ceased. The eggs were then washed and brought to 2% v/v suspension in ASW. A 1.5% v/v suspension was also used in a few cases, when females spawned too few eggs for the cross design on a given day. Eggs were stored at 5–9°C. Mantle tissue was removed from the female and stored in DMSO at 4°C for genotyping.

### (c) Non-competitive crosses

A non-competitive cross is defined as a pairing of the eggs of one female with the sperm of one male in two replicate series, with each series being composed of six different dilutions of sperm, each mixed with an equal concentration of eggs (for a total of twelve scintillation vials per cross). The purpose of these crosses was to combine (in all possible pairwise combinations of males with each female) sperm and eggs together in the absence of sperm competition. In this manner we generated fertilization curves for each non-competitive cross, providing a measure of gamete compatibility [11], [17]. This measure is generated from a regression model that is used to estimate the predicted number of offspring that would be sired by a male at the sperm concentrations tested in the competitive crosses.

Vials were filled with 4 ml ASW and into each vial 0.5 ml of 2% egg suspension was pipetted using a wide-bore tip. Six ten-fold sperm dilutions of dry sperm were prepared ranging from 10-1 to 10-6. To each of the egg suspensions 0.1 ml of sperm dilution was added. The suspensions were swirled to mix and then incubated at 8°C for 6–8 hours, the approximate time at which cleaving embryos reach the 8 to 32-cell stage. As there is no obvious gross morphological change to the egg that occurs at fertilization in *Mytilus* (such as a fertilization membrane), cleaving is used as a proxy for fertilization. Samples were then fixed with 0.5 ml of 37% formaldehyde. One hundred eggs per replicate were scored as either cleaving or not cleaving under a compound microscope.

### (d) Competitive crosses

The purpose of the competitive crosses was to combine eggs with sperm of two males, and therefore allow any potential competitive interactions between these sperm to occur just prior to fertilization. Competition is detected as a deviation of the paternity ratio (the ratio of offspring sired by male A to that sired by male B) from that predicted in crosses between the same female and each of the males individually assayed under non-competitive conditions. Conspecific sperm precedence then is defined as a higher observed conspecific to heterospecific offspring ratio for a cross, compared to that predicted from the non-competitive crosses. Since species identity in these mussels cannot be easily established morphologically prior to setting up the cross, individuals were chosen from locations known to show bias in the relative frequency of *M. trossulus* and *M. edulis* to increase the odds that these competitions were between heterospecific and conspecific males. Each female (most of which were *M. edulis*) was exposed to nine competitive crosses using six males, three from the *M. trossulus*-biased population and three from the *M. edulis*-biased population. Eggs were exposed to each sperm combination at two volumetric ratios, 10∶1 and 1∶1 *M. trossulus*:*M. edulis* sperm (hereafter, 10∶1 T:E and 1∶1 T:E). The 10∶1 T:E ratio was chosen because homospecific sperm are typically 10 to 1000 times more compatible with eggs than are heterospecific sperm in non-competitive assays [11], [12]; this is an attempt to allow the heterospecific male enough fertilizations to permit detection of deviations. Mixtures at the 1∶1 T:E ratio would be predicted to allow too few eggs to be fertilized by heterospecific sperm for sufficient scope to detect deviations; nevertheless, these were performed in case their analysis was deemed necessary after outcomes were known.

To begin, 36 scintillation vials were filled with 4.0 ml of ASW, and with 0.5 ml of the 2% egg/ASW suspension from one female using a large bore tipped pipette. The process was repeated for three females on each day of spawning, for a total of 108 scintillation vials per day. Six males were assigned to each female, three from each of the two collection locations. Each male from the first location was competed with each male from the second location, for a total of 9 competitions per female. Mixing of sperm to create the “competitive mixture” was accomplished just prior to adding the mixture to the eggs, to ensure that sperm were well mixed and that there were no effects of order of addition to the eggs. Three replicates of each of the two sperm ratios (1∶1, 10∶1) were mixed immediately before being added to the egg suspensions, since sperm of free-spawners begin to swim immediately after exposure to seawater and viability is known to decline rapidly after dilution [18].

Once all incubations were mixed, they were incubated for approximately 8 hours, after which eggs were washed and settled. The ASW with very concentrated non-viable sperm was decanted from the scintillation vials and 10 ml of fresh ASW was added to each vial. The eggs were then incubated for another 64 hours at 6–9°C. All vials were checked throughout the incubation period for larvae and monitored for the onset of the D-stage, or prodissoconch I [19]. When these were observed, at approximately 72 hours, all larvae were fixed. The larvae were reared to the D-stage to ensure they were sufficiently large for successful genotyping, while at the same time reducing, as much as possible, the contribution from any post-zygotic incompatibilities acting during larval development. The larvae were fixed with 7 ml Modified Salt Ethanol (MSE; [20]) and the contents were settled. The liquid was decanted, and a final 4 ml of MSE was added to the vials.

### (e) Genetic analysis

Mantle tissue from mussels used in crosses was extracted using the DNeasy Kit (Qiagen, Valencia, CA) and genotyped using three species diagnostic codominant PCR-based markers: Glu5′ [21], Mal I [21] and ITS [22]. Individuals that, at all three loci, were homozygous for either *M. trossulus* or *M. edulis* alleles were considered “pure” species for the purpose of the experiment. Upon genotyping, those competitive crosses found to involve a pair of males, one heterospecific and one homospecific with the female (i.e. one “pure” *M. trossulus* male and one “pure” *M. edulis* at all 3 markers) were identified for further analysis. Larvae from the competitive crosses were then extracted using a modification of the method by Simpson et al. [23], and genotyped using the Glu5′ marker, which provided unambiguous classification of larvae as the product of homospecific or heterospecific fertilization (Glu 5′ homozygous and heterozygous genotypes, respectively; [21]). Fifty individuals were genotyped from each of the three replicates of the competitive crosses). Glu5′ genotyping was performed on 1.5% agarose gels.

### (f) Statistical analysis

The relationship between the proportion of fertilized eggs and sperm concentration for each of the non-competitive crosses was determined using a logit transformation as described by McCartney and Lessios [17]. Linear regression of logit-transformed estimates of the proportion of fertilized eggs as dependent and log-transformed sperm concentration as independent variables were analyzed by linear regression for each of the non-competitive crosses, while the number of progeny predicted to be sired at the sperm concentration of interest was determined from the regressions for each male. The predicted proportion of offspring sired was then calculated by dividing this number, for each male, by the sum of the values for the two males. It is important to note that this value assumes no CSP—it simply considers the sperm concentrations of the two males in the mixture, and the estimated progeny sired for each male at those concentrations, under non-competitive conditions.

Contingency table analysis was then used to determine whether outcomes were significantly different among the three replicates [24]. When they were not, the replicate counts were pooled to determine the total observed number of offspring for each of the males. The predicted share of offspring sired for each male was then compared with the observed numbers of offspring sired using G-tests for goodness of fit, and the Williams correction [24], [25]. In addition, because a number of the predicted values were small, a two-tailed exact binomial test was performed for each cross [24]. All results were evaluated for significance using Bonferroni corrected α-values.

## Results

### (a) Parental genotypes and sperm counts

A total of fifteen competitive crosses, out of the ninety performed, represented competitions between heterospecific and homospecific males. The 3 females involved in these crosses— females A, E, and H, were combined in six, three and three competition crosses, respectively. All females were *M. edulis*, so this study did not examine competitions involving *M. trossulus* as the egg parent. In our work over 9 years in eastern Maine, we found that *M. trossulus* females spawned in response to induction (from early May and throughout the spawning season) much less frequently than *M. edulis* females, and in the present study, too few *M. trossulus* females spawned to provide combinations needed to study CSP in this direction. The dry sperm concentrations for each of the *M. trossulus* males in these crosses were similar, ranging from 2.34×10^9^ to 4.36×10^9^ sperm/ml. In comparison, the *M. edulis* dry sperm concentrations were more variable. While most of the *M. edulis* males ranged between 1.36×10^8^ to 3.68×10^9^, one male had a concentration of 1.79×10^10^ sperm/ml. Together this resulted in greater realized *M. trossulus*:*M. edulis* sperm concentration ratios than the intended 10∶1 volumetric ratio. One possible reason for these discrepancies is the method of extracting sperm, since the volume of fluids from the mantle cavity extracted along with sperm is difficult to control. When checked, no effects on sperm fertility were found (correlation between dry sperm concentration and log F20 values was not significant: n = 18, *r* = 0.084, P = 0.742).

### (b) Non-competitive crosses

Analysis of variance showed that the regression coefficients were significant in all cases, with the exception of one trial, which, with its three associated competition crosses, was eliminated from further analysis, leaving a total of twelve competitive crosses (Table 1). Crosses with female E indicated a strong block to fertilization by heterospecific sperm, while the two other females appeared to be less blocked. Heterospecific crosses of female E yielded an F_20_ (the concentration of sperm required to fertilize 20% of eggs) greater than the highest sperm concentration tested, whereas the F_20_ values from the heterospecific crosses of female A and H were several magnitudes lower (meaning these crosses were far more compatible). This variation among females is typical. Both Rawson et al. [11] and Slaughter et al. [12] found that fertilization of *M. edulis* eggs by *M. trossulus* sperm varied over a similar range, across a larger sample of *M. edulis* females. In the present study, we found that female H showed such high compatibility that the F_20_ values for heterospecific and homospecific males nearly overlapped.

**Table 1:** Linear regression analyses of results from non-competitive crosses.

Female	Male	F_20_	*a*	*b*	*R^2^*	*F*
A	T1	4.95×10^5^	−5.56	0.32	0.8	40.75***
	T2	6.70×10^5^	−6.64	0.39	0.85	56.33***
	E1	7.53×10^1^	−4.45	0.69	0.81	42.91***
	E2	1.46×10^1^	−2.85	0.55	0.88	75.90***
	E3	6.37×10^0^	−5.47	0.51	0.88	71.71***
E	T3†	2.96×10^13^	−5.36	0.13	0.48	9.16*
	T4	-	−5.01	0.08	0.24	3.22
	E4	1.06×10^2^	−3.86	0.53	0.83	47.65***
	E5	7.61×10^3^	−5.89	0.5	0.86	63.46***
	E6	5.27×10^2^	−4.07	0.43	0.75	29.61***
H	T5	3.86×10^3^	−3.68	0.28	0.83	49.73***
	E7	5.62×10^1^	−3.59	0.52	0.86	62.49***
	E8	1.69×10^3^	−5.3	0.53	0.8	40.48***
	E9	2.48×10^3^	−3.59	0.28	0.71	24.00***

All females are *M. edulis* and males are *M. edulis* (E) and *M. trossulus* (T).

† The value is provided because the regression is significant, but F20 in this cross is biologically unrealistic (i.e. it exceeds the concentration of dry sperm).

F_20_ are in sperm/ml, *a* =  the intercept, *b* =  the regression coefficient, and *F* =  the F-value (*P<0.05; **P<0.01; ***P<0.001).

### (c) Competitive crosses

**Figure 1:**
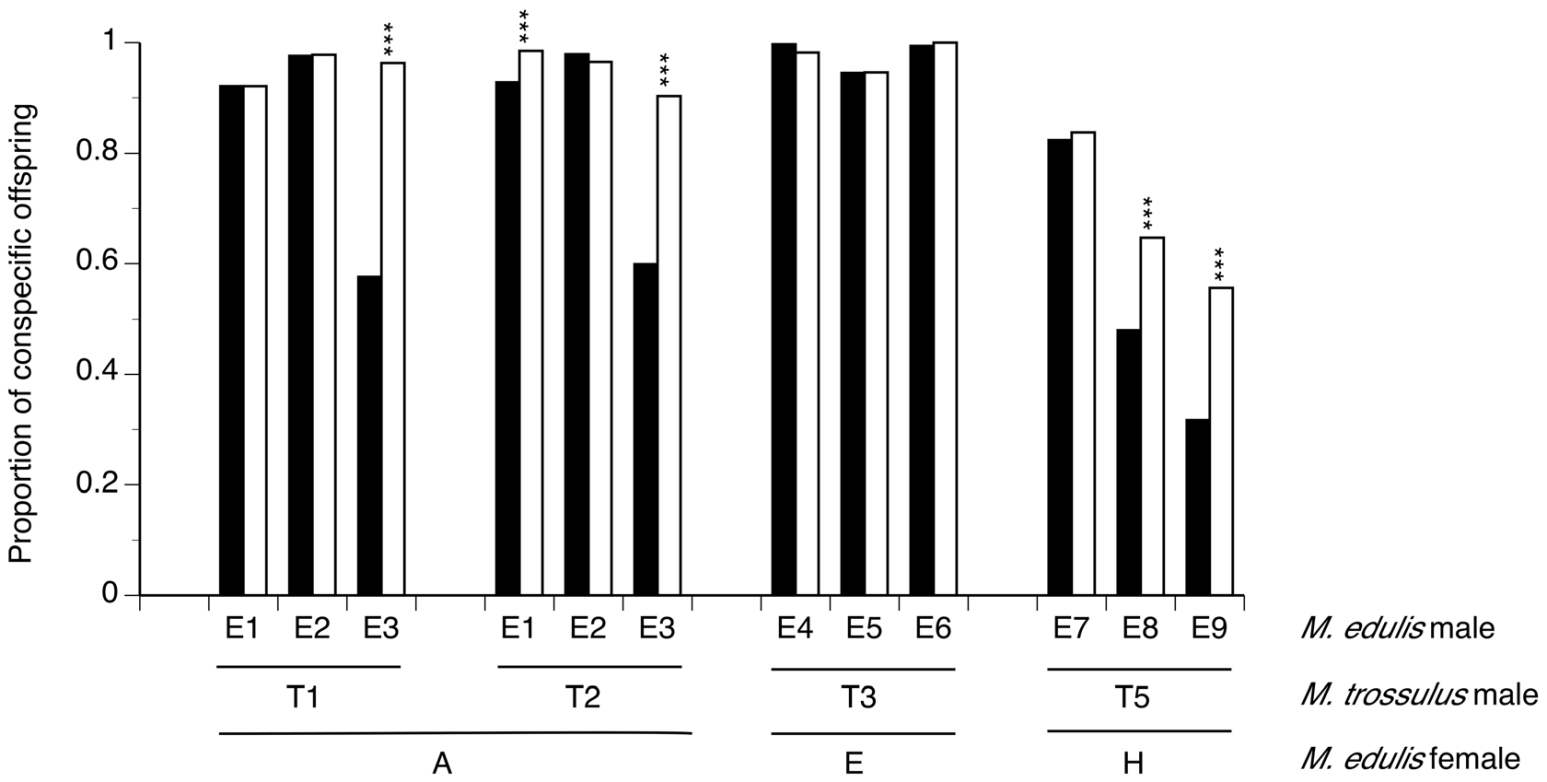
Patterns of CSP variation. Predicted proportion (filled bars) and observed proportion (open bars) of conspecific offspring sired in each competition cross. Asterisks mark cases of CSP that are significant (G-test): ***P<0.001.

**Figure 2:**
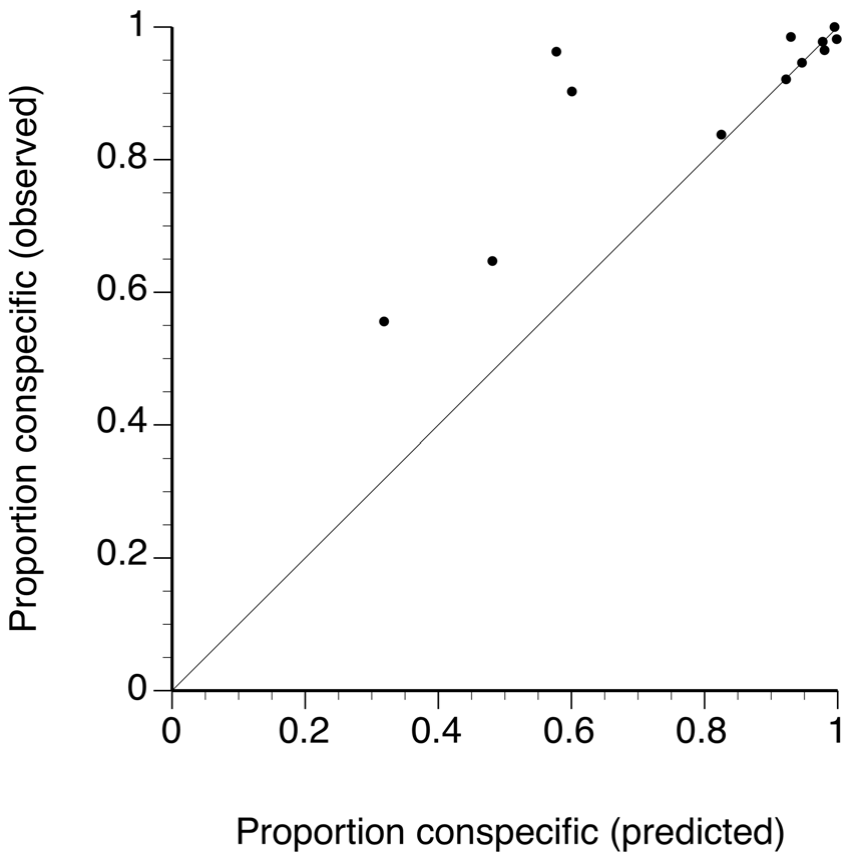
CSP is greatest in crosses that are compatible between species. Plot of observed against predicted proportion of conspecific offspring sired in each competitive cross. The solid line indicates no difference (i.e. no competition). The five points substantially above the line show significant CSP, and 4 of these occurred in cases in which <70% of offspring were predicted to be sired by the conspecific male in the absence of competition.

Five of the 12 competition crosses showed CSP, with significantly more conspecific offspring produced than predicted under the null hypothesis of no sperm competition (Table 2, Fig. 1). One cross showed a slight decrease in conspecific offspring that was significant (only by the binomial exact test), and in six crosses, departures from the null were absent, or small and not significant. Each of the crosses with no CSP showed high cross-species incompatibility in crosses with no competition, while those with CSP were more compatible (expected heterospecific paternity proportions mostly in the range of <0.10 and>0.40, respectively: Fig. 2). This shows that CSP was strongest when sperm was mixed with eggs from the most compatible females. Whether this pattern relates to the mechanism of CSP or whether it relates to the power of our assay for detection (or both) is not clear. The results indicate that CSP would strengthen prezygotic isolation under competitive conditions, however it does not completely eliminate heterospecific fertilization of the eggs of compatible females.

**Table 2:** Results from competition crosses.

*M. edulis* female	*M. edulis* male	*M. trossulus* male	Proportion of *M. edulis* sperm	Observed number of *M. edulis* larvae	Expected number of *M. edulis* larvae	Observed number of *M. trossulus* larvae	Expected number of *M. trossulus* larvae	G	Binomial Probability
A	E1	T1	0.005	129	129	11	11	0	
	E2	T1	0.024	131	131	3	2	0	
	E3	T1	0.009	129	77	5	57	108.4***	***
	E1	T2	0.005	132	124	2	10	10.03***	**
	E2	T2	0.024	136	138	5	3	1.133	
	E3	T2	0.009	131	87	14	58	67.20***	***
E	E4	T3	0.136	213	216	4	1	5.120	***
	E5	T3	0.044	176	176	10	10	0	
	E6	T3	0.434	140	139	0	1	2.000	
H	E7	T5	0.024	93	91	18	20	0.250	
	E8	T5	0.031	141	104	77	113	24.01***	***
	E9	T5	0.039	74	42	59	91	32.57***	***

Significance of G-statistics and exact binomial test results (*P<0.05; **P<0.01; ***P<0.001) were assessed using Bonferroni-adjusted alpha values.

While the present data set is too small to investigate them in any detail, the results do suggest the presence of interactive effects between pairs of males (and perhaps between males and females). With *M. edulis* female A, while the sperm of M. edulis male E3 showed CSP when mixed with sperm from M. trossulus male T1 and when mixed with sperm from T2, the sperm of M. edulis male E1, only showed CSP when mixed with sperm from *M. trossulus* male T2; this male showed no precedence over T1, even though it should have been detectable (Table 2; Fig. 1). And both *M. edulis* males E8 and E9 showed CSP over male T5 in assays with eggs of female H, but there was no CSP of male E7 over male T5.

## Discussion

Our results demonstrate conspecific sperm precedence in *M. edulis* females in a direct comparison of non-competitive and competitive crossing experiments with *M. trossulus*. Using sperm concentration ratios that are biased towards *M. trossulus*, we were able to show that when heterospecific *M. trossulus* sperm and conspecific *M. edulis* sperm compete for fertilizations of *M. edulis* eggs, the number of heterospecific offspring can be significantly lower than what is predicted in the absence of competition. Though CSP was clearly observed, it was not found in all crosses. Of the twelve crosses analyzed, a significant reduction in the number of heterospecific offspring was observed in five. In the remaining seven, one cross showed a slight but significant increase in the number of heterospecific offspring compared to the predictions, and six crosses showed no significant change in the number of offspring predicted and the number observed. Overall, CSP appeared to be strongest in the most compatible heterospecific crosses, which are also the most relevant to the likely role of CSP in natural spawns. Taken together, our observations add to previous findings that gametic incompatibility can be an effective barrier to hybridization [1]–[3], [7], [8]. Our findings show that CSP can strengthen gametic isolation, and lend support to earlier assertions that the evolution of CSP may be an important step in speciation after secondary contact.

This study adds to the evidence for the roles that gamete incompatibility and CSP play in reproductive isolation and speciation between the 3 species in the *M. edulis* complex. Allopatric speciation split North Atlantic *M. edulis* from North Pacific *M. trossulus* ancestors sometime after 3.5 MYA (when the Bering Strait opened to allow invasion of the Atlantic, and more recently closed during glaciations), and about 2 MYA, *M. galloprovincialis* diverged, due to geographic isolation in the Mediterranean, from *M. edulis* (reviewed in [26]). Rawson et al. [11] showed that incompatibility is much stronger between *M. edulis* and *M. trossulus* than it is between the more closely related sister pair *M. edulis* and *M. galloprovincialis* [27], and suggested that this reproductive barrier helps account for much lower frequencies of introgressed hybrids between the former species pair. This finding is consistent with work in sea urchins that shows that gamete incompatibility increases with genetic distance—although its evolution is episodic and not continuous [28]. Since CSP may result from mechanisms that are distinct from those that cause non-competitive gamete incompatibility (see below), its evolution should be considered separately; particularly relevant is the time course of CSP evolution, and how it relates to progress towards speciation in hybrid zones. There is some evidence to suggest that CSP exists between *M. edulis* and *M. galloprovincialis* [27], which would represent an exception to the finding that CSP is uncommon in unimodal hybrid zones [6]. Gametic incompatibility between these two species was not reported in non-competitive crosses, and only when gametes from both species were mixed was “assortative fertilization” demonstrated [27]. The study design in Bierne et al. [27] created ambiguity in ascribing effects to CSP or to fertilization incompatibility (although the authors favor CSP). If CSP exists between *M. edulis* and *M. galloprovincialis*, we would conclude that its presence alone is not sufficient to shift a hybrid zone towards bimodality, a stage approaching complete speciation [6]. More studies on CSP between species in the *M. edulis* complex would help clarify this issue. They would also improve understanding of CSP evolution in relation to the biogeographic history of the complex and the genetics of *Mytilus* hybrid zones, and the role CSP plays in limiting hybridization.

Our results do clearly demonstrate the incompleteness of gametic isolation between the two actively hybridizing species in the western Atlantic. When sperm of heterospecific males dominated mixtures, and when females showed high compatibility with these males, hybrid offspring were frequently produced, even in crosses where CSP was observed. In our assays, CSP reduced conspecific fertilizations by 5.7–38.7%, but several heterospecific fertilizations remained— 1.5 to 44.4% of all offspring were hybrids. Rawson et al. [11] suggested compatible females to be a likely route through which hybridization occurs in natural populations. In the light of all results to date, this hypothesis is plausible, considering the overlapping reproductive seasons of the two species [13], the lack of spatial segregation of adults (personal observations), and from the present results—the inability of CSP to eliminate hybrid fertilizations.

To enhance our power of detecting CSP, we used sperm mixtures that were greatly biased toward higher concentrations of heterospecific sperm. How relevant might this be to natural spawns? A partial answer comes from earlier studies of the fertilization ecology of other marine invertebrates. These studies have demonstrated that proximity of spawning individuals, population density, and water flow can all greatly influence fertilization success under natural conditions. With spawning sea urchins Levitan and coworkers [28]–[30] demonstrated a clear increase in fertilization success in sea urchins with aggregation, decreased flow velocity, and when individuals were in downstream and central positions in relation to other aggregating individuals. Yund and McCartney [31] showed evidence for intraspecific sperm competition in two sessile, colonial marine invertebrates—a bryozoan and an ascidian. They found that the fertilization success of a male placed a fixed distance from a female colony was greatly reduced by sperm competition with a competing male placed between them. Our present findings of CSP between two blue mussel species suggest some potential interactions between local density and distance between mates and sperm precedence in natural mussel beds. For example, a conspecific male may still have a fertilization advantage even if it is farther away from a female than a heterospecific male. Furthermore, hybrid offspring are likely only to be formed when the local density of heterospecific sperm greatly exceeds conspecific sperm. While this outcome is not surprising, the present findings provide guidance for future work on the fertilization ecology of blue mussels. Studies of gamete interactions between mussels under more natural conditions of water flow are of particular interest. One approach would use controlled flow in laboratory flumes, in which density and proximity of individuals of both species could both be manipulated.

In non-competitive crosses, compatibility varied greatly among female *M. edulis*, as in other studies [11], [12] but the relationship of compatibility to the strength of CSP is not clear. In each of the females we see some crosses resulting in CSP, and some having no significant precedence. When competed in mixtures with *M. trossulus* male T1 for fertilization of eggs of *M. edulis* female A, *M. edulis* male E3 shows strong and highly significant CSP but not males E1 and E2 (Fig. 2). Interestingly, when male T2 was tested on the same female, the pattern was similar across these three *M. edulis* males, again with male E3 showing by far the strongest CSP, although in this case, CSP was also found for male E1. The degree of CSP also does not appear to be simply a result of how compatible (or fertile) a male is in non-competitive, homospecific crosses. Male E3 was the most compatible (lowest F_20_: Table 1) of the 3 males tested for compatibility with female A in conspecific crosses, and it does show the greatest level of CSP. However, males E8 and E9 are less compatible with female H in conspecific non-competitive crosses, but showed higher CSP than male E7. Geyer and Palumbi [8] suggested that a combination of sperm competition (male-male interactions between sperm) and egg-sperm compatibility may ultimately determine the intensity of CSP. It is important to distinguish these two properties, as the mechanisms responsible for sperm competitive success and non-competitive fertility may be different. Our results suggest they are and, while based on few trials so far, also suggest that the males with the highest competitive success may not be those that show the highest fertility under non-competitive conditions.

In *Mytilus*, CSP is mechanistically most likely to involve steps of sperm-egg interaction. Steps identified cytologically include induction of the acrosome reaction [32], binding of sperm to carbohydrates on the egg vitelline envelope (VE; [33]), and VE dissolution [34]—each known to be species specific steps in other invertebrate systems. The most well-studied in mollusks is VE dissolution, which in abalone is controlled by sperm acrosomal VE lysins binding to an egg surface receptor known as VERL ([reviewed in [35]). The rate of VE dissolution depends on the quantity of VE lysin applied to eggs, and this rate is greater in homospecific VE/lysin combinations than it is in heterospecific combinations [36]. Species-differential rates of VE dissolution that generate modest differences in fertilization rates in non-competitive conditions could be amplified under sperm competition, leading to CSP. *Mytilus* has acrosomal lysins [37] and while their species differential function has not been studied biochemically, cDNAs for two *Mytilus* lysin loci are known to evolve under positive selection [37]–[39]; a hallmark of the abalone lysins [35]. *Mytilus* egg VE proteins that serve as the cognate receptor for lysins are unknown, moreover, and therefore we can only speculate upon whether CSP in blue mussels may be controlled by sperm or egg, or both.

A recent study by Miranda et al. [40] examined a number of reproductive barriers between *M. edulis* and *M. trossulus*. Though this study and ours are complementary there are several differences between them. Miranda et al. [40] performed reciprocal competitive and non-competitive crosses, however, they incubated the resulting larvae for 20 days before genotyping for parentage. We performed non-reciprocal competitive and non-competitive crosses, and incubated for 3 days. According to a study by Toro et al. [41], once larvae are incubated past three days post-zygotic barriers begin to cause a decrease in hybrid larval survival. Up to day 3, they observed no differential mortality between conspecific and heterospecific larvae. Thus the findings by Miranda et al. of a significantly lower number of heterospecific larvae may not reflect solely an effect of CSP, but instead possible CSP in addition to other post-zygotic barriers. Our results demonstrate CSP when *M. edulis* and *M. trosslulus* sperm compete to fertilize *M. edulis* eggs. Since we did not examine the reciprocal cross, and given the ambiguities in the methods of Miranda et al. [40], we would conclude that it is not yet clear whether CSP might occur when *M. trossulus* is the egg parent. Future work to examine CSP in the reciprocal cross would be valuable, as Miranda et al. [40] have demonstrated substantial variation in non-competitive compatibility of *M. trossulus* females. In Newfoundland, *M. trossulus* females are induced to spawn at higher frequencies [40] than in eastern Maine, so it may be that the northern end of the hybrid zone is a better setting for this work.

Though their results are inconclusive for CSP, Miranda et al. [40] do provide clear evidence for post-zygotic barriers. Miranda et al. have demonstrated an early acting post-zygotic barrier, which affects larvae between the third and tenth day post fertilization. This coincides with the onset of feeding in the larvae, a good candidate for a life stage transition in which differential mortality is likely to be manifesting [19]. The presence of strong post-mating pre-zygotic barriers and strong early acting post-zygotic barriers would suggest a smaller role for late-acting post-zygotic isolating mechanisms. Jiggins and Mallet [6] suggest a necessity for some post-zygotic barrier in a bimodal hybrid zone, but state that the pre-zygotic barriers are key. It may be then that the early acting post-zygotic barriers observed here are sufficient to prevent the recombinational shuffling of “mate recognition systems,” thus reducing the necessity for strong environmental effects on hybrid fitness, such as appear to be operating in the Baltic *M. edulis*/*M. trossulus* hybrid zone [26].

Jiggins and Mallet [6] also suggested that reinforcement should be present in bimodal hybrid zones. This has not, however, been demonstrated in the western Atlantic *Mytilus* hybrid zone. A previous study by Slaughter et al. [12] examined evidence for character displacement in gamete incompatibility, a signal of reinforcement, between populations of *M. edulis* that were sympatric with *M. trossulus*, compared to allopatric populations. They did not observe reproductive character displacement but rather a trend in the opposite direction, with *M. edulis* females being more compatible within the hybrid zone than outside of it. This does not, however, mean that reinforcement is not occurring; it may be acting instead on a different pre-zygotic barrier [42]. A study examining CSP in allopatric and sympatric populations may yield the pattern of reproductive character displacement that is predicted by the bimodal distribution of hybrid genotypes within the hybrid zone, that Jiggins and Mallet [6] suggest should provide the conditions driving reinforcement. Coyne and Orr [2] argue that reinforcement should act first on female gametes. They reason that since sperm are unlikely to encounter more than one egg in their lifespan (i.e. they cannot detach once they collide with an egg) selection is not likely to favor choosiness on the part of sperm. This would suggest "choice” is more likely to evolve in eggs, and that eggs should be the target for the pattern of character displacement associated with reinforcement. If CSP is a product of selection on eggs, we might therefore expect it to be a target for reinforcement selection.

Within the *Mytilus* hybrid zone it is becoming increasingly clear that despite strong pre- and post-zygotic isolating barriers to reproduction, the hybrid population within the northwestern Atlantic *Mytilus* hybrid zone is being maintained, most likely, through highly compatible heterospecific fertilizations that result in few, but fertile offspring. Now several studies [11], [12], [40] including the present one have documented broad variability in both intra- and interspecific fertilization success, and have observed high compatibility in some heterospecific crosses. Previous studies examining CSP in marine invertebrates have been conducted on species that have few or no natural hybrids or in systems where other pre-mating or post-zygotic barriers are strongly influential [7], [8]. Geyer and Palumbi [8] found strong CSP between sympatric species of sea urchins, however, there is no evidence of natural hybridization between the species. Harper and Hart [7] likewise identified CSP between sympatric species of Asterias sea stars, however, there is no consensus as to whether the species hybridize naturally. Our results describe CSP in a system where reproductive isolation is incomplete, pre-mating barriers are limited and evidence for strong post-zygotic barriers is equivocal [26]. Taken together, these observations suggest that in this *Mytilus* hybrid zone, gametic isolation, both competitive and non-competitive, is playing a large role in reproductively isolating these two closely related, sympatric populations.
